# Left Ventricular Rotational Mechanics in Tanzanian Children with Sickle Cell Disease

**DOI:** 10.1016/j.echo.2014.11.014

**Published:** 2015-03

**Authors:** Michael V. Di Maria, Hao H. Hsu, Ghassan Al-Naami, Jeanine Gruenwald, K. Scott Kirby, Fenella J. Kirkham, Sharon E. Cox, Adel K. Younoszai

**Affiliations:** a University of Colorado School of Medicine, Children's Hospital Colorado, Aurora, Colorado; b Division of Pediatric Cardiology, Children's Hospital & Medical Center, University of Nebraska Medical Center, Omaha, Nebraska; c Fort McMurray, Alberta, Canada; d Department of Neurosciences, UCL Institute of Child Health, London, United Kingdom; e Muhimbili Wellcome Programme, Muhimbili University of Health & Allied Sciences, Dar es Salaam, Tanzania; f The London School of Hygiene and Tropical Medicine, London, United Kingdom

**Keywords:** Sickle cell disease, Sickle cell disease, Left ventricular torsion, Left ventricular torsion, Hb, Hemoglobin, LV, Left ventricular, MSC, Muhimbili Sickle Cohort, SCD, Sickle cell disease

## Abstract

**Background:**

Sickle cell disease (SCD) is a common inherited hemoglobinopathy. Adults with SCD manifest both systolic and diastolic cardiac dysfunction, though the age of onset of dysfunction has not been defined. Left ventricular (LV) rotational mechanics have not been studied in children with SCD. The aim of this study was to investigate whether cardiac rotational mechanics differed between children with SCD and age-matched controls.

**Methods:**

Basal and apical LV short-axis images were acquired prospectively in 213 patients with SCD (mean age, 14.1 ± 2.6 years) and 49 controls (mean age, 13.3 ± 2.8 years) from the Muhimbili Sickle Cohort in Dar es Salaam, Tanzania. The magnitude of basal and apical rotation, net twist angle, torsion, and untwist rate were obtained by two-dimensional speckle-tracking. The timing of events was normalized to aortic valve closure.

**Results:**

Mean basal rotation was significantly lower in patients with SCD compared with controls (*P* = .012), although no difference was observed in apical rotation (*P* = .37). No statistically significant differences in torsion or net twist angle were detected. Rotation rate at the apex (*P* = .001) and base (*P* = .0004) were significantly slower in subjects with SCD compared with controls. Mean peak untwisting rate was also significantly slower in patients with SCD (*P* = .006). No associations were found between hemoglobin concentration and apical rotation, basal rotation, net twist, and torsion.

**Conclusion:**

This study demonstrates alterations in LV rotational mechanics in children with SCD, including lower basal rotation, peak differential twist, and untwist rate. These abnormalities denote subclinical changes in LV systolic and diastolic performance in children with SCD. Future work may reveal an association between rotational metrics and long-term patient outcomes.

Sickle cell disease (SCD) is a common hemoglobinopathy, the sequelae of which can be life threatening.^1^ Patients with SCD are at risk for left ventricular (LV) systolic dysfunction.^2-6^ Previous studies have also demonstrated that this population is also susceptible to diastolic dysfunction,^7-9^ and some suggest that this diastolic dysfunction plays a role in the development of pulmonary hypertension.^2,10^ One proposed mechanism for the development of cardiac dysfunction is microvascular occlusion in the coronary circulation as a result of red blood cell sickling, resulting in chronic, indolent myocardial ischemia.^11^ In addition, chronic anemia leads to a high cardiac output state and LV volume overload. Over time, this may cause LV dilation, which may also adversely affect LV function.

Conventional echocardiographic measures of LV function, such as shortening fraction and ejection fraction, have not been demonstrably different when patients with SCD and unaffected individuals have been compared^7,10^; however, decreased LV peak systolic strain in subjects with SCD suggests that conventional methods of evaluating LV function by measuring chamber size, such as shortening fraction, are not precise enough to detect early dysfunction.^10^ LV rotation, net twist angle (twist), and torsion, a means of quantifying the rotational movement of the myocardium during systole,^12^ have been studied in other disease states and have demonstrated an early decline in LV systolic performance.^13-15^ LV torsion has not been previously studied in pediatric patients with SCD. We hypothesized that there would be significant differences in rotational metrics between individuals with SCD and healthy age-matched controls. The aim of this study was to evaluate cardiac rotational mechanics in patients with SCD, compared with age-matched, unaffected control subjects.

## Methods

A nested case-control study was undertaken, including study participants with SCD and unaffected control patients from the Muhimbili Sickle Cohort (MSC) in Dar es Salaam, Tanzania.

### Study Population

Cases were recruited prospectively from patients enrolled in the MSC and controls from children who had attended the clinic for sickle screening and were determined not to have SCD. Inclusion criteria included age between 9 and 19 years and, for patients with SCD, sickle status confirmed by hemoglobin (Hb) electrophoresis and high-performance liquid chromatography, while controls without SCD had the HbAS or HbAA phenotype by electrophoresis. Blood pressure was measured twice after 5 min of rest using an automated digital device (PRO 400 V2 Dinamap; GE Healthcare, Little Chalfont, United Kingdom) and the mean calculated. Axial temperature, height, and weight were recorded, and a clinical examination was performed to exclude acute complications. Exclusion criteria included history of blood transfusion within the previous 2 months, symptoms of sickle crisis within the previous 2 weeks, febrile illness within the 7 days before echocardiography, fever or signs of acute illness on the day of echocardiography, and known or echocardiographically identified hemodynamically significant congenital heart disease.

The study was approved in the institutional review board of Children's Hospital Colorado (Reference No. COMIRB protocol #10-0030) and by the Muhimbili University of Health & Allied Sciences Research and Publications Committee (Reference No. MU/RP/AEC/Vol.XIIV/01). Written informed consent was obtained from all study participants.

### Definitions

**Figure 1:**
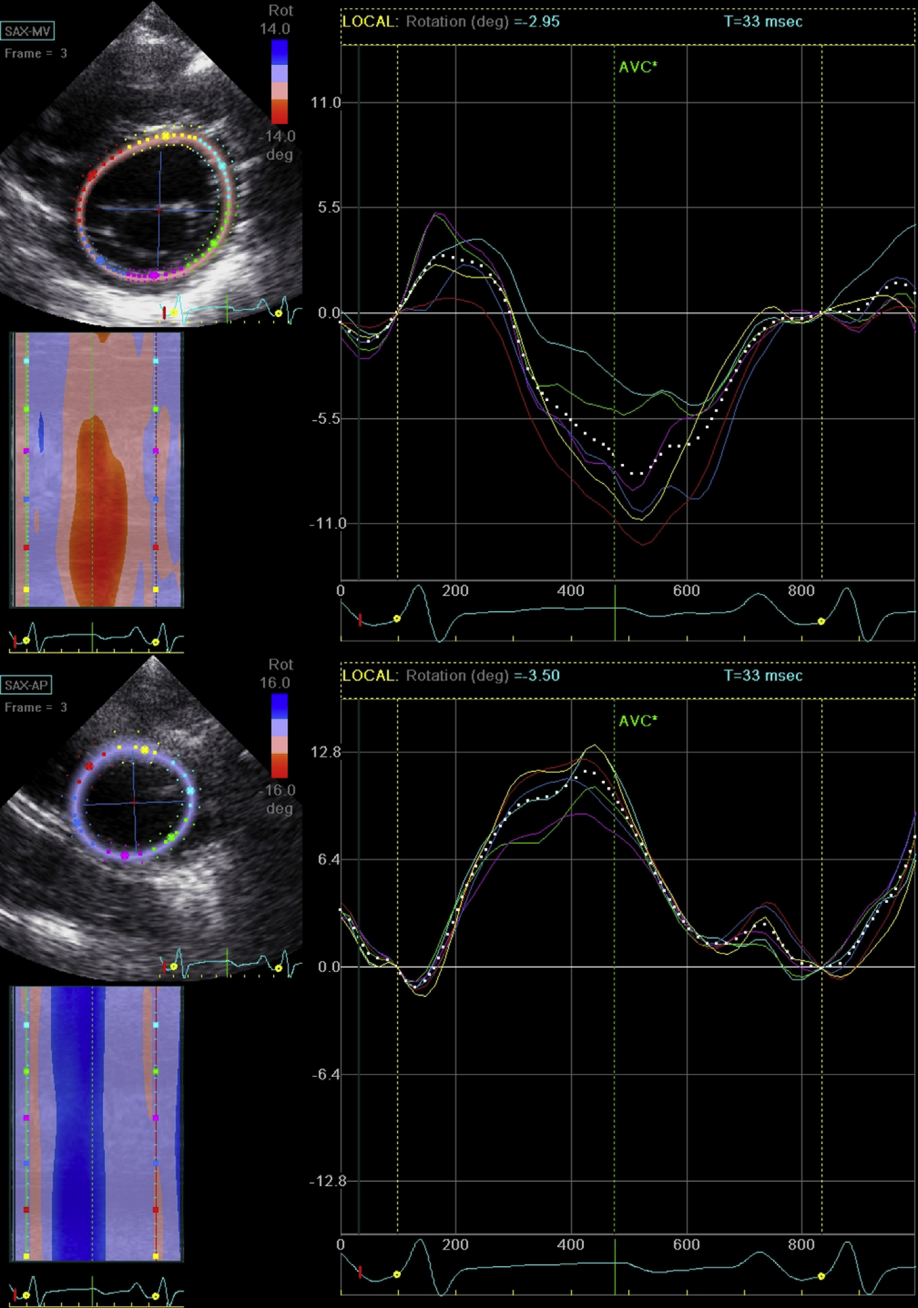
Assessment of rotational mechanics using two-dimensional speckle-tracking echocardiography. Representative rotation-time plots for a subject with SCD. Basal and apical short-axis planes were obtained as shown, and speckle-tracking analysis was performed. Peak global rotation on each image was measured (*white dots*) with reference to the electrocardiogram below (the Q wave is marked by a *yellow dot*) and aortic valve closure (indicated by a vertical *green line*). *SCD*, Sickle cell disease.

Rotation was defined as circumferential myocardial motion around the centroid, or long axis, of the ventricle.^12^ By convention, rotation is said to be positive in the counterclockwise direction when viewed from the apex of the left ventricle and vice versa. As depicted in Figure 1, a basal short-axis plane used to determine twist was obtained just below the mitral valve annulus, where muscle appears in the plane of insonation throughout the cardiac cycle. The apical short-axis plane used was defined as a short-axis plane just above the apex, where the LV lumen can be seen throughout the cardiac cycle. Net twist angle was defined as the instantaneous difference in rotation between the apex and the base at the time of aortic valve closure, or end-systole.^12^ Peak differential twist is the maximum difference in rotation between the apex and base, independent of time. Torsion was defined as net twist divided by the length of the left ventricle at end-diastole to account for heart size. Untwisting is defined as the difference between apical recoil and basal recoil. The timing of these events is described both in milliseconds and as a percentage of systole, with the onset of systole defined as the initial deflection of the Q wave on the surface electrocardiogram and end-systole defined as aortic valve closure.

### Echocardiography

For each patient, measurements of LV chamber size and fractional shortening were made from short-axis basal images. From the apical window, LV peak systolic mitral annular velocity (LV S′), LV peak early diastolic mitral annular velocity (LV E′), early diastolic mitral inflow (LV E), duration of late diastolic mitral inflow (mitral valve a-wave duration), and duration of the late diastolic a wave in pulmonary vein inflow (pulmonary vein a-wave duration) were made. The LV myocardial performance index was also calculated from mitral inflow and aortic outflow spectral Doppler patterns.^16^

Short-axis images of the base and apex of the left ventricle were obtained using the VividQ platform (GE Healthcare) and an M4S, 5S, or 7S probe, depending on patient characteristics and image quality. Frame rates of >75 and <125 frames/sec were accepted. An acquisition of five consecutive beats was obtained. An apical view was also obtained for the purposes of measuring end-diastolic LV length and performing spectral Doppler measurement of mitral inflow and aortic outflow. End-diastolic LV length was measured from the apical four-chamber view as the distance from the mitral valve to the apex of the left ventricle.

Commercially available software (EchoPAC version 113, revision 0.4; GE Healthcare) was used to perform speckle-tracking analysis. A single cardiac cycle was isolated for the base, and a region of interest was identified. The magnitude and timing of basal rotation was calculated. The process was repeated for the short-axis apical image. Net twist was calculated as the difference between apical and basal rotation. Torsion was calculated by dividing the net twist by LV end-diastolic length. This process was repeated two additional times and an average over three cardiac cycles was calculated.

### Laboratory Data

Hb phenotype was determined by alkaline Hb electrophoresis (Helena; Sunderland, United Kingdom) and by quantification of Hb fractions, including HbF, by high-performance liquid chromatography (β-Thalassemia Short Program, VARIANT analyzer; BioRad, Hercules, CA). In subjects with SCD, the most recent steady-state Hb, total bilirubin, conjugated bilirubin, and lactate dehydrogenase levels were obtained from MSC records (steady state defined as absence of pain or fever, malaria rapid test negative, and with no recorded hospital admission or blood transfusion within 30 days). Full blood counts were performed using an automated cell counter (Pentra 60; Horiba ABX, Kyoto, Japan). Biochemical tests were performed using an automated chemistry analyzer (Cobas Mira [Roche, New York, NY] or Abbott Architect [Abbott Diagnostics, New York, NY]).

### Statistical Analysis

SAS version 9.2 (SAS Institute Inc, Cary, NC) was used to perform statistical analyses. Continuous variables are presented as mean ± SD or median (interquartile range), and categorical variables are presented as proportions. The Student independent two-sample *t* test was used to compare mean values between two patient groups. Simple linear regression was used to evaluate associations between independent variables and a continuous, dependent variable. Pearson correlation was used to evaluate associations between measures of rotational mechanics and Hb or laboratory measures of hemolysis in the children with SCD. A *P* value < .05 was determined to be the threshold for statistical significance. Analyses were compared when the five children with HbSB+ were included or excluded from the patients with SCD and no material differences were observed, and therefore all reported analyses include both the HbSS and HbSB+ cases.

### Interobserver and Intraobserver Variability

Eleven consecutive patients were evaluated separately by two independent observers (H.H.H. and G.A.), who evaluated net twist, torsion, twist rate, and peak untwisting rate in a blinded fashion. To reflect the methodology used in this study, each measurement was made on what the investigator felt was the best cardiac cycle among the five that were acquired. A single observer (H.H.H.) performed serial analysis of the same 11 patients 2 months later using the same methodology to determine intraobserver variability. Analysis included calculation of intraclass correlation coefficients of variation.

## Results

**Figure 2:**
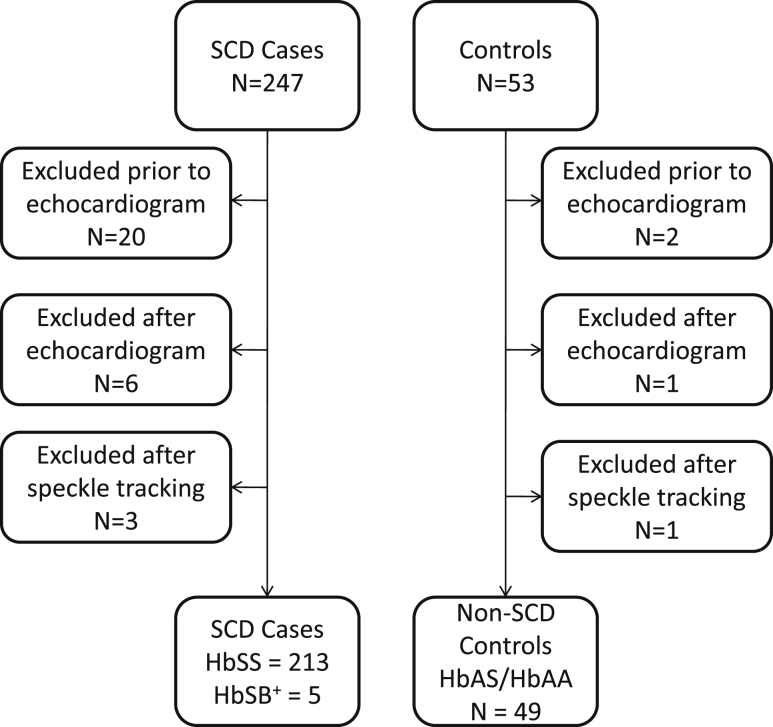
Flowchart depicting the numbers of patients with SCD and controls and the reasons for exclusion from the study. *SCD*, Sickle cell disease.

Three hundred subjects were recruited into the study. On the day of the echocardiographic assessment, 22 subjects were excluded before echocardiography because they were unwell, while seven subjects were excluded after echocardiography because of minor structural cardiac abnormalities. Four additional subjects were excluded after speckle-tracking analysis because of poor image quality. Thus a total of 262 participants were included in the present analysis, consisting of 213 patients and 49 unaffected controls (see Figure 2).

Baseline demographic and conventional echocardiographic data are summarized in Table 1. There was no significant difference in age or gender composition between the two groups. Patients with SCD had higher heart rates (*P* = .0042), lower mean Hb levels (*P* < .0001), and lower systolic (*P* < .0001) and diastolic (*P* = .0002) blood pressures. The SCD group also had lower body mass index *Z* scores (*P* < .0001) and lower height-for-age *Z* scores (*P* < .0001). The SCD group had significantly greater LV dilation in both the long- and short-axis dimensions, as assessed by LV end-diastolic dimension *Z* score and LV length (*P* < .0001 for both comparisons). The LV sphericity index was significantly lower in the SCD group (*P* = .006), indicating that the left ventricle more closely approximated a sphere, which has a sphericity index of 1. No difference was observed in fractional shortening (*P* = .16) or in tissue Doppler measurements of LV function. However, the LV myocardial performance index was significantly different in the SCD group (0.336 ± 0.12 vs 0.368 ± 0.12, *P* = .03).

The results for intraobserver and interobserver variability are summarized in Table 2. There was good agreement between both serial measurements by the same investigator and blinded measurements by different, sequential observers.

Table 3 reports the rotational mechanics of patients with SCD compared with controls without SCD. Mean peak apical rotation was not different between the two groups, but peak basal rotation was significantly lower in the SCD group (−3.88 ± 2.3° vs −3.13 ± 2.5°). There was no significant difference in net twist, but controls had significantly higher peak differential twist (9.55 ± 3.93° vs 8.2 ± 3.25°) and peak normalized differential twist (1.42 ± 0.56°/cm vs 1.13 ± 0.46°/cm). There was no significant difference in mean torsion, but controls had a significantly higher torsion rate (12.06 ± 4.5°/sec/cm vs 9.53 ± 3.5°/sec/cm). Peak untwist rate was significantly slower in subjects with SCD compared with controls (*P* = .006).

There was no significant association between age and net twist in the SCD group. Similarly, there was no association between net twist and mean arterial pressure. We also assessed relationships between the most clinically relevant measures of LV rotational mechanics (apical rotation, basal rotation, net twist, and torsion) and Hb (*n* = 216), B-type natriuretic peptide (*n* = 106), and lactate dehydrogenase (*n* = 199), in patients with SCD only, but none were significant (data not shown). We also performed a quartile analysis with regard to torsion, comparing Hb, lactate dehydrogenase, and B-type natriuretic peptide between the highest and lowest quartiles; there was no significant difference in any biomarker between those with the most robust torsion and the least.

## Discussion

This study reveals significant differences in LV rotational mechanics between Tanzanian children with SCD and age-matched controls. To our knowledge, this is the first study in children with SCD to evaluate rotational mechanics and to detect early declines in these measures of systolic and diastolic LV performance.

Other investigators have demonstrated reductions in both LV and right ventricular strain in both adults and children with SCD. Sengupta *et al*.^10^ evaluated young adults with SCD during sickle crisis and demonstrated attenuation of LV systolic deformation, most notably longitudinal strain; however, only subepicardial circumferential strain remained significantly decreased on evaluation after recovery from crisis. Decreased right ventricular longitudinal strain has also been demonstrated in children with SCD.^17^ These findings corroborate the hypothesis that this population develops subclinical myocardial dysfunction long before any changes in radial shortening are detectable.

Lower peak untwisting rate correlates with diastolic dysfunction in other populations.^8,9,18^ The slower peak untwisting rate we observed in patients with SCD compared with controls supports the idea that the development of LV diastolic dysfunction is an early event in children with SCD. In adults with SCD, conventional markers of diastolic function are abnormal, primarily tissue Doppler and mitral inflow Doppler, as shown by Caldas *et al*.^4^ Our population of children with SCD had normal tissue velocity, early mitral inflow peak velocity, and a normal ratio of peak inflow to tissue velocity, providing additional supportive evidence for the ability of peak untwisting rate to detect early diastolic dysfunction.

Children with SCD have been shown to have LV dilation in proportion to the degree of anemia, though with a preserved shortening fraction.^19^ Unexpectedly, our data showed no associations between Hb or markers of hemolysis (unconjugated bilirubin and lactate dehydrogenase) and apical rotation, basal rotation, twist, or torsion. This may indicate that chronic anemia itself, rather than the direct effects of hemolysis, may be a primary cause of cardiac dysfunction. As others have, we speculate that other etiologies, such as myocardial ischemia related to vaso-occlusive events,^11^ may play a larger role in the development in myocardial damage and dysfunction. Ischemic myocardial injury has been noted histologically on autopsies of patients with SCD,^20^ though this mechanism remains hypothetical. A more recent study presented histologic findings of myocardial fibrosis in adult patients with SCD, demonstrating considerable heterogeneity within the cohort.^21^ Risk factors for fibrosis, rate of progression, and correlation with outcomes remain unstudied in children with SCD. Iron-mediated cardiomyopathy is a leading cause of death in patients with hemoglobinopathies^22^; however, our cohort does not receive frequent blood transfusions, so this is not a consideration.

Further support for the notion that the patients with SCD under study are manifesting early ventricular dysfunction is provided by the findings of other investigations of the effect of increased preload, and hence end-diastolic diameter, on LV torsion. Dong *et al*.^23^ evaluated the effect of increasing end-diastolic volume on rotational mechanics in isolated, perfused canine hearts and showed that increasing preload resulted in an increase in torsion. A study in which eight young, healthy human subjects underwent evaluation of LV torsion before and after administration of normal saline infusion showed a 33% increase in LV torsion, driven primarily by an increase in apical rotation.^24^ Others have evaluated the effect of sphericity index on LV rotation and twist in controls and subjects with dilated cardiomyopathy,^25^ describing a parabolic relationship between both LV apical rotation and twist versus sphericity index. In healthy individuals, peak apical rotation and twist occurred at a sphericity index of approximately 2, with higher and lower values associated with less rotation and twist.^25^ They hypothesized that the changes in twist are related to myofiber orientation. Interestingly, with increasing sphericity index, basal rotation remained unchanged in controls as well as individuals with dilated cardiomyopathy. In our study, the subjects with SCD who had significantly larger end-diastolic volumes and lower sphericity indices showed no change in apical rotation or LV torsion but a decrease in basal rotation. We hypothesize that the difference in sphericity index between the two groups in our study, although statistically different, is not large enough alone to result in detectable changes in apical rotation or twist. We hypothesize that our SCD cohort's decrease in mean basal rotation suggests that children with SCD may have novel pattern of dysfunction requiring further study to characterize.

Ahmad *et al*.^26^ used three-dimensional speckle-tracking to evaluate myocardial deformation in a group of 28 adults with SCD and normal controls and observed significant differences in global longitudinal strain but no difference in twist or torsion. Our expectation would be that adults and children with the same disease would manifest similar patterns of dysfunction. However, we speculate that adult patients residing in the United States may experience a different progression of SCD-related pathophysiologic changes compared with Tanzanian children. However, differences in technique concerning three-dimensional versus two-dimensional speckle-tracking could also explain this disparity, making comparisons between these populations problematic. Preliminary investigations into the comparability between two-dimensional and three-dimensional assessment of twist mechanics in an animal model showed promise.^27^

### Limitations

As subjects were recruited from a regional care center for SCD, sonographers and study interpreters were not blinded to the disease state of each group.

Longitudinal data are required to understand the development and progression of systolic and diastolic dysfunction over time and any associations with morbidity or mortality of SCD.

## Conclusions

This study demonstrates that despite normal fractional shortening, subclinical systolic and diastolic cardiac dysfunction is present in children with SCD, as assessed by rotational mechanics. Further studies are needed to evaluate the onset and progression of alterations in ventricular mechanics in children with SCD, as these indices may be predictors of adverse outcome later in life.

## Figures and Tables

**Table 1:** Baseline anthropometric and conventional echocardiographic data

Demographic variable	Controls (*n* = 49)		Patients with SCD (*n* = 218)		*P*
	*n*	Mean ± SD or count (%)	*n*	Mean ± SD or count (%)	
Age (y)	49	13.3 ± 2.8	218	14.1 ± 2.6	.07
Male gender	49	26 (52%)	218	122 (55%)	.20
Heart rate (beats/min)	49	80.5 ± 16.4	218	86.1 ± 11.1	.0042
Hb (g/dL)	49	11.6 ± 1.7	218	7.2 ± 1.1	<.0001
Systolic blood pressure (mm Hg)	49	116 ± 13.2	218	109 ± 10.2	<.0001
Diastolic blood pressure (mm Hg)	49	68 ± 8.4	218	64 ± 6.0	.0002
Body mass index *Z* score	49	−0.57 ± 1.29	218	−1.5 ± 1.2	<.0001
Height-for-age *Z* score	49	−0.96 ± 1.3	218	−2.26 ± 1.14	<.0001
LV dimension					
LV end-diastolic diameter *Z* score	49	-0.42 ± 1.1	217	2.25 ± 1.4	<.0001
LV end-diastolic length (cm)	49	6.7 ± 0.7	217	7.3 ± 0.6	<.0001
LV sphericity index	49	1.62 ± 0.2	216	1.53 ± 0.1	.0006
LV function					
Fractional shortening (%)	49	37.91 ± 4.67	217	38.14 ± 4.26	.16
LV myocardial performance index	47	0.34 ± 0.11	205	0.37 ± 0.12	.03
LV S′ velocity (cm/sec)	46	10.18 ± 2.08	213	10.01 ± 1.84	.39
LV E′ velocity (cm/sec)	47	19.86 ± 3.48	210	19.93 ± 3.38	.50
Mitral E-wave velocity (m/sec)	49	1.00 ± 0.17	216	1.07 ± 0.17	.50
Mitral valve inflow E wave to E′ ratio	47	5.15 ± 1.24	209	5.54 ± 1.51	.34
A-wave duration ratio (mitral inflow/pulmonary A reversal)	20	1.22 ± 0.26	77	1.26 ± 0.40	.35

*LV*, Left ventricular. *Z* scores were generated using reference data from healthy children at Boston Children's Hospital (Boston, MA).

**Table 2:** Assessment of interobserver and intraobserver variability

Variable	Intraclass correlation coefficient
Interobserver	
Torsion	0.95
Net twist	0.95
Torsion rate	0.75
Untwist rate	0.77
Intraobserver	
Torsion	0.98
Net twist	0.98
Torsion rate	0.81
Untwist rate	0.90

**Table 3:** Rotational mechanics of subjects with SCD versus age-matched controls

Variable	Controls (*n* = 49)		Patients with SCD (*n* = 218)		*P*
	*n*	Mean ± SD	*n*	Mean ± SD	
Apical rotation (°)	48	5.6 ± 2.7	200	5.0 ± 2.4	.37
Basal rotation (°)	47	−3.9 ± 2.3	208	−3.1 ± 2.5	.012
Net twist (°)	46	7.7 ± 4.1	198	6.9 ± 3.8	.09
Peak differential twist (°)	47	9.5 ± 3.9	198	8.2 ± 3.2	.016
Peak normalized differential twist (°/cm)	47	1.4 ± 0.6	198	1.1 ± 0.5	.017
Torsion (°/cm)	46	1.1 ± 0.6	198	0.9 ± 0.5	.12
Torsion rate (°/sec/cm)	46	12.1 ± 4.5	198	9.5 ± 3.5	.0002
Untwist rate (°/sec)	46	−94.5 ± 37.1	198	−83.0 ± 32.2	.006
Normalized untwist rate (°/sec/cm)	46	−14.2 ± 5.8	198	−11.4 ± 4.6	.004
Time to peak apical rotation (% systole)	48	86.4 ± 22.0	200	89.6 ± 25.9	.23
Time to peak basal rotation (% systole)	47	125.9 ± 26.2	208	116.6 ± 19.7	.53
Time to peak torsion (% systole)	46	102.6 ± 7.9	198	105.9 ± 9.9	.16
Time to peak untwist rate (% systole)	46	129.1 ± 10.2	198	131.3 ± 10.3	.16
Apical rotation rate (°/sec)	48	70.7 ± 28.3	200	57.7 ± 23.0	.00127
Normalized apical rotation rate (°/sec/cm)	48	10.6 ± 4.4	200	7.9 ± 3.3	.00065
Basal rotation rate (°/sec)	47	−73.8 ± 24.4	208	−64.90 ± 21.1	.00048
Normalized basal rotation rate (°/sec/cm)	47	−11.0 ± 3.7	206	−8.95 ± 3.0	.00039
Net twist rate (°/sec)	46	80.5 ± 30.7	199	69.12 ± 24.8	.0004

*SCD*, Sickle cell disease.
